# Trace amine-associated receptor 1 agonist reduces aggression in brain serotonin-deficient tryptophan hydroxylase 2 knockout rats

**DOI:** 10.3389/fpsyt.2024.1484925

**Published:** 2024-12-19

**Authors:** Ilya S. Zhukov, Yazen Alnefeesi, Natalya A. Krotova, Vsevolod V. Nemets, Konstantin A. Demin, Marina N. Karpenko, Evgeny A. Budygin, Evgeny V. Kanov, Allan V. Kalueff, Petr D. Shabanov, Michael Bader, Natalia Alenina, Raul R. Gainetdinov

**Affiliations:** ^1^ Institute of Translational Biomedicine, St. Petersburg State University, St. Petersburg, Russia; ^2^ Institute of Experimental Medicine, Saint Petersburg, Russia; ^3^ National Research University Higher School of Economics, Moscow, Russia; ^4^ Department of Neurobiology, Sirius University of Science and Technology, Sirius, Russia; ^5^ St. Petersburg University Hospital, St. Petersburg State University, St. Petersburg, Russia; ^6^ Department of Biosciences and Bioinformatics, School of Science, Xi’an Jiaotong-Liverpool University, Suzhou, China; ^7^ Suzhou Municipal Key Laboratory of Neurobiology and Cell Signaling, School of Science, Xi’an Jiaotong-Liverpool University, Suzhou, China; ^8^ Max Delbrück Center for Molecular Medicine in the Helmholtz Association (MDC), Berlin, Germany

**Keywords:** serotonin, TPH2, 5HT, TAAR1, aggression, RO5263397, TAAR1 agonist, social dominance

## Abstract

**Introduction:**

Aggression and self-harm disproportionately occur in youths preoccupied with social status tracking. These pathological conditions are linked to a serotonin (5-HT) deficit in the brain. Ablation of 5-HT biosynthesis by tryptophan hydroxylase 2 knockout (TPH2-KO) increases aggression in rodents. Remarkably, deletion of the trace amine-associated receptor 1 (TAAR1) results in the same consequences. Unlike the nuanced dynamics of social status cues in young people, the social ranks of rats mainly advance when they dominate larger opponents in combat.

**Methods:**

This study explored whether the potent TAAR1 agonist RO5263397 reduces aggression caused by 5-HT depletion, and whether social rank advancement motivates this aggression. The resident-intruder paradigm was applied with larger and smaller intruders to evaluate whether social rank advancement motivates aggressive behaviors in TPH2-KO rats.

**Results:**

When a smaller intruder was introduced, 5-HT-deficient rats did not differ from wild type littermates. However, when the intruders were larger, the mutants extended their aggressive efforts, refusing to submit. Importantly, RO5263397 selectively abolished this abnormal form of aggression in TPH2-KO rats.

**Discussion:**

Results supported social rank advancement as the main incentive. These data also suggest that TAAR1 is a promising target for the development of new treatments for aggression; independent data also support this conclusion.

## 1 Introduction

Aggression and self-harm co-occur in many psychiatric patients with bipolar disorder, depression and other mental illnesses. Selective serotonin reuptake inhibitors (SSRIs) are commonly prescribed in these pathological conditions (1, 2). However, some studies suggest that SSRIs can trigger suicidality and uncharacteristic acts of violence (3, 4). Although fraught with controversy, substantial evidence suggests that aggression and self-harm are rare side effects of SSRIs, especially evident in youths (5). Leaflets for SSRIs warn of treatment-emergent suicidality in *adolescents and young adults*. Violent crime and self-harm are particularly common in people aged < 25 years (6, 7). This is no coincidence (8), since this age group is more sensitive to social status cues (9). Strong evidence suggests that both aggression and self-harm are tightly linked with social status tracking (10–12).

The SSRI class acts by increasing the synaptic level of serotonin (5-HT) (13), which affects many aspects of animal social hierarchies (14, 15) and the stress response (16). The synthesis of 5-HT from the essential amino acid tryptophan is mediated by two spatially segregated isoforms of the enzyme tryptophan hydroxylase (TPH); one is peripheral and the other is central (17). The central TPH2 isoform is predominantly expressed in neurons originating in the brainstem, with projections to the frontal cortex, amygdala, and hippocampus. In rodents, genetic deletion of TPH2 (TPH2-KO) causes increased aggression, social deficits, and altered anxiety-like behaviors (18–23). Both humans and non-human primates exhibit links between 5-HT, aggression, and self-harm (9, 24–26). This corroborates prior indications that the role of 5-HT in violence is conserved across species.

The trace amine-associated receptor 1 (TAAR1) is the most studied member of the TAAR family. It participates in reward and limbic networks and is expressed in several brain regions (78), including the medial prefrontal cortex (mPFC). Serotonergic signaling in this area is known to critically influence the top-down regulation of aggression (27). Initially, TAAR1-KO rodents were proposed as models of schizophrenia (28), showing not only enhanced responses to amphetamine and deficits in sensorimotor gating but also altered sleep-wake cycles and increased impulsivity (29, 30). The TAAR1 agonists RO6889450 (Ralmitaront) and SEP-363856 (Ulotaront) are now under active clinical development for schizophrenia treatment (31). Nevertheless, preclinical studies suggest that TAAR1 could mediate multiple neuropsychiatric effects, exerting antipsychotic, antidepressant, procognitive, anti-obsessive, anti-addictive, and sleep-modulating actions (32, 33).

Importantly, it was previously demonstrated that TAAR1-KO promotes aggression, increases 5-HT in the prefrontal cortex, affects sexual motivation, and reduces grooming in mice (34). These results are particularly interesting when compared to the TPH2-KO phenotype. The present study thus aimed to test whether TAAR1 activation reduces the aggression caused by 5-HT depletion, and whether this aggression is linked to social hierarchies as it is in young people (11, 12). Given the centrality of rat aggression in this study, an explanation of rat social aggression is due. Male rats form social dominance hierarchies on the basis of age and body weight (35). When rats fight, the aggression typically stops when one animal signals social defeat through submissive posture and the cessation of retaliatory efforts. The animals usually stop attacking when their opponents submit in this way and most confrontations are brief (<60 sec is common). It is thus abnormal for a much smaller rat to persevere, or for a much larger rat to continue attacking after winning.

In this study, the resident-intruder paradigm was applied to represent for residents (i.e., TPH2-KO or WT littermates) opportunities to advance in social rank (i.e., bigger intruders) and lack thereof (i.e., smaller intruders). Results suggest that the highly aggressive behavior of TPH2-KO rats is motivated by an exaggerated drive to advance in the social dominance hierarchy. Most importantly, the TAAR1-specific agonist RO5263397 abolishes this type of aggression, and independent data now replicate this under different conditions (36).

## 2 Materials and methods

### 2.1 Ethics statement

All animal studies were carried out in accordance with the guidelines of the Ministry of Health of the Russian Federation, the Federation of European Laboratory Animal Science Associations (FELASA), and the Russian Laboratory Animal Science Association (RusLASA) for the welfare of laboratory animals. All experiments were approved by the Saint Petersburg State University Ethical Committee for Animal Research (approval 131-03-1 of 13.03.2022).

### 2.2 Subjects

**Figure 1:**
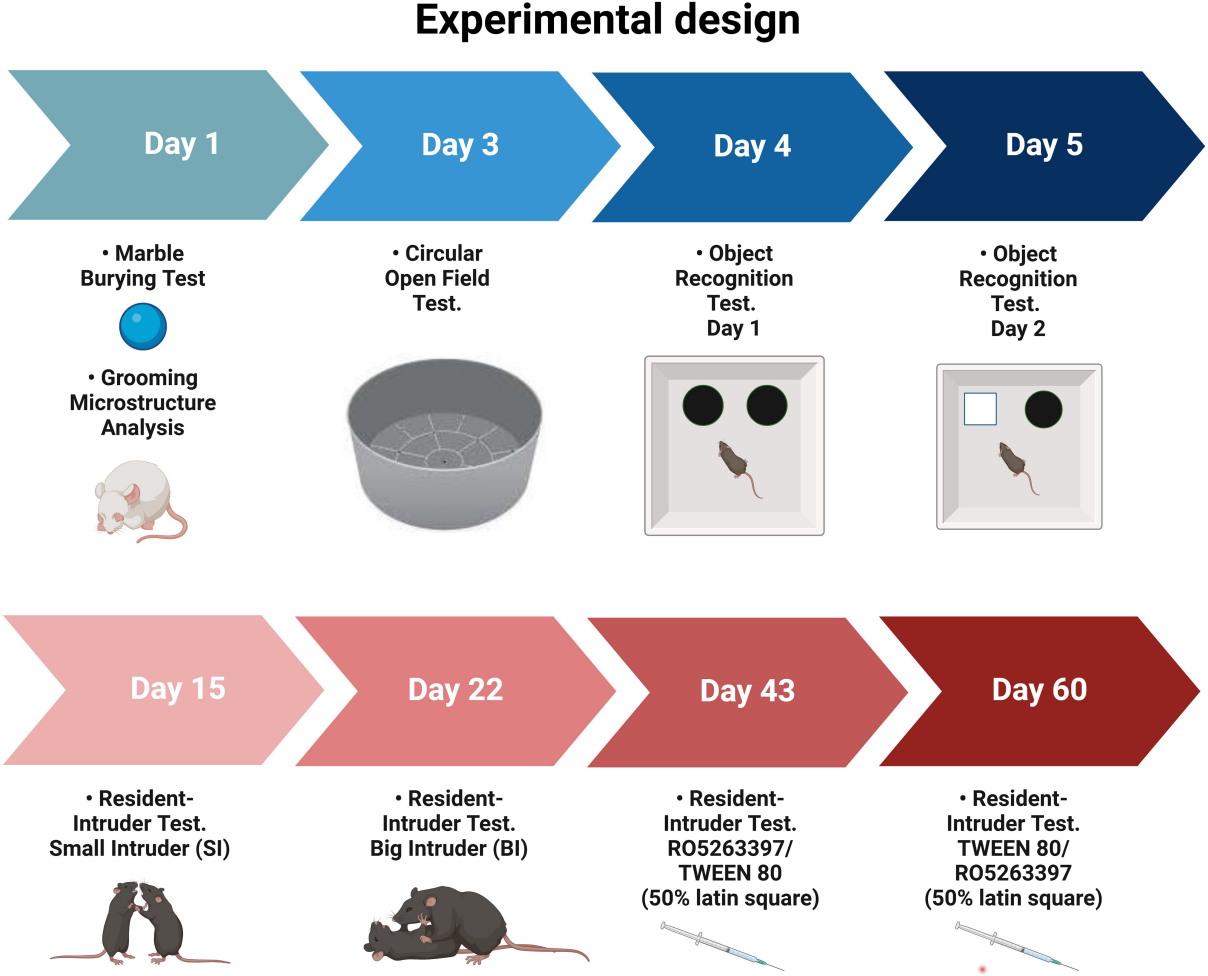
Experimental design of comparative analysis between WT and TPH2-KO rats (n = 8 per group).

Middle-aged adult wild-type (WT) and TPH2-deficient (TPH2-KO, (79) male rats on Dark Agouti background (35 weeks old, n = 8 per genotype, mean weight WT = 312 g [SD = 22], TPH2-KO = 317 g [SD = 17]) were derived by crossing animals heterozygous for the TPH2 mutation and genotyped as previously described (37). Animals were maintained under standard lab conditions (room temperature and humidity 21 ± 5°C and 40–70%, respectively) with food and water *ad libitum*, under artificial light-dark cycle (lights on 10:00, lights off 22:00). All experiments were conducted during the light phase from 15:00 to 22:00 at an illuminance of 265 lux throughout. The rats were acclimated to the experimental room for at least 1 h prior to behavioral testing. A scheme of the experimental design is shown in **Figure 1**. All rats were genotyped before and after the experiments.

### 2.3 Drugs

A TAAR1 agonist (S)-4-(3-Fluoro-2-methylphenyl)-4,5-dihydrooxazol-2-amine (RO5263397; CAS#: 1357266-05-7), synthesized at F. Hoffmann-La Roche Ltd (Basel, Switzerland), was chosen for its high intrinsic efficacy (76 ± 13%) and specificity at the rat TAAR1 receptor, as evidenced by the lack of effects in TAAR1 knockout animals (30, 38). Its half-life (T_1/2_) of 2.6 h (39) was also conducive to experimental purposes. Fresh solutions of 5 mg/ml RO5263397 in 10% buffered Tween 80 were prepared on the same day of every experiment. Intraperitoneal injections were administered at doses of 1 ml/kg body weight (i.e., 5 mg/kg) 10 min before the start of the resident-intruder interaction. The dose test sequences were based on a within-subjects Latin Square design (i.e., each animal was tested with both an active dose and vehicle) with at least 2 weeks between the tests, to ensure washout and stress recovery.

### 2.4 Behavioral assays

The Open Field Test (OFT) was used to assess the locomotor and exploratory activity of rats. The circular arena was made of gray plastic with a diameter of 67 cm, and included 13 uniform holes of 1 cm diameter in the floor. The test subject was placed in the center of the arena, after which its natural exploratory behavior was recorded for 10 min using Noldus EthoVision XT Version 11.5 (Noldus IT, Wageningen, Netherlands) followed by manual scoring by highly experienced experimenters blinded to genotype. The endpoints recorded were: locomotor activity (distance traveled, cm), freezing (sec), sniffing (sec), grooming (sec), number of rearings (n), and hole exploration (sec). To eliminate the confounding effects of variable olfactory cues, the apparatus was cleaned with a 3% hydrogen peroxide solution before each trial for all tests herein.

The OFT also served as the adaptation session (Day 0) for a subsequent Object Recognition Test (ORT) conducted in the same arena over the next two days (40). On Day 1, single animals were released into the testing arena facing two identical objects (black cylinders). To quantify recognition memory, the next sessions on Day 2 only differed in that the familiar object on the left was substituted with a novel one (a white pyramid of approximately equal mass). Data were recorded similar to the OFT analyses, scoring the duration of exploration of right and left objects (s), grooming (s), movement (s), and rest/inactivity (s). Only active exploration (i.e., sniffing or rearing on an object) was counted, and rest/inactivity near objects was not included in exploration time.

The Marble Burying Test (MBT) evaluates compulsive-like behavior in rodents (41). Twenty marbles were placed on the surface of clean bedding (~5 cm layer, 400 ± 15 g per cage) in polycarbonate rat cages of standard dimensions, and the number of marbles buried (n) during a 30-min session was counted. Due to the bigger size of rats (than mice for which the test was initially designed), only fully buried marbles not visible from the top were counted.

The Grooming Test (GT) was applied to quantify both basic self-grooming metrics in addition to grooming microstructure (42, 43). Briefly, rats were individually placed in a transparent glass cylindrical jar (20 cm in diameter, 45 cm in height) and their grooming was recorded for 10 min using Apple iPhone 12 video camera (Apple Inc., Cupertino, USA). Recorded grooming behavior was then manually scored to ascertain: total grooming bouts (n), rostral grooming bouts (n), caudal grooming bouts (n), as well as grooming bouts (n) specific to the paw, face, head, body and tail. To further analyze grooming microstructure, the number of grooming transitions (n) between different body parts (e.g., nose to head, head to tail) were also noted here. Any transition between grooming stages that violated normal cephalo-caudal progression (paws > face/nose > head > body > tail/anogenital) was considered incorrect (this included skipping body parts), allowing the calculation of the percent correct transitions (i.e., [correct/total]*100%). The number of transitions within each cephalo-caudal progression was noted and ethograms were generated to represent microstructural sequences as done previously (44).

An adapted and extended resident-intruder paradigm (34, 45) was used here to evaluate territorial aggression in the context of differential dominance. Female rats require more elaborate protocols and smaller intruders to avoid confounds and floor effects in this assay (46); the study thus used male rats for the sake of simplicity. Isolated TPH2-KO and WT male rats were observed for 10 min with a socialized (i.e., group-housed) intruder. Male Wistar rats (white coat) were used as intruders to ensure reliable distinction from residents (dark coat). Bedding material of the home cage (590х380х200 mm) was not changed for 1 week before the experiment to ensure an undisturbed sense of territory. Different intruders were used after every 3-4 confrontations. The videos were manually scored to ascertain: seconds spent in social interaction (anogenital sniffing, tail sniffing, body sniffing, nose sniffing), non-social exploration (rearing, wall stand, burying, sniffing), and aggression parameters (attack latency, lateral threat, upright posture, clinch attack, keep down, chase), as well as locomotor endpoints such as movement (sec), and rest/inactivity (sec). Mounting behavior was evaluated by recording the number of mountings (n) and intromission time (sec).

The experiment consisted of two phases. First, the socialized 50% smaller weight intruder (SI, Wistars, WT male, 8 weeks) was placed in front of the TPH2-KO or WT male resident to assess aggression. Following 1-week habituation to fresh bedding, the experiment was repeated with socialized 30% bigger weight intruders (BI, Wistars, WT male, 15 weeks). Three weeks later, the experiment was repeated once more with residents under a RO5263397 pretreatment and intruders from the BI group (full datasets and video samples are presented in **Supplementary Materials**). Aggression towards smaller intruders who give postural signals of social defeat is redundant because they do not pose a threat to the larger resident’s territory or social dominance status. Conversely, aggression towards larger intruders signifies an attempt to advance one’s social dominance status. Rats generally form social hierarchies on the basis of age and body mass (35). This study varied the age and body mass of intruders, effectively modelling differential social ranks (see **Supplementary Material** for individual weights of all resident-intruder pairs).

### 2.5 Statistical analyses and visualization

Analyses and visualization were performed with GraphPadPrism 8.0 (GraphPad Software, USA). Data are expressed as the mean ± standard error of the mean (SEM) or the median and interquartile range. The non-parametric Mann–Whitney U test (α = 0.05; two-tailed) was used to compare the WT and TPH2-KO groups for baseline behavioral assays. Results from the extended resident-intruder paradigm were analyzed by two-way repeated measures ANOVA (Factors: F*^Genotype^* is WT vs. TPH2-KO; F*^Intruder^* is bigger vs. smaller intruders; F*^Drug^* is RO5263397 vs. vehicle [i.e., TWEEN80]) followed by Dunnett’s *post-hoc* test (α = 0.05; two-tailed). Colored visualization of statistical differences: WT vs. WT = * (blue), KO vs. KO = * (orange), WT vs. KO = * (violet). Exhaustive statistical reports are available in **Supplementary Materials Table 2**. Additional visualizations were conducted with the R radar-plot package, and a licensed account on BioRender.com.

## 3 Results

### 3.1 TPH2-KO alters exploratory behavior and laterality

**Figure 2:**
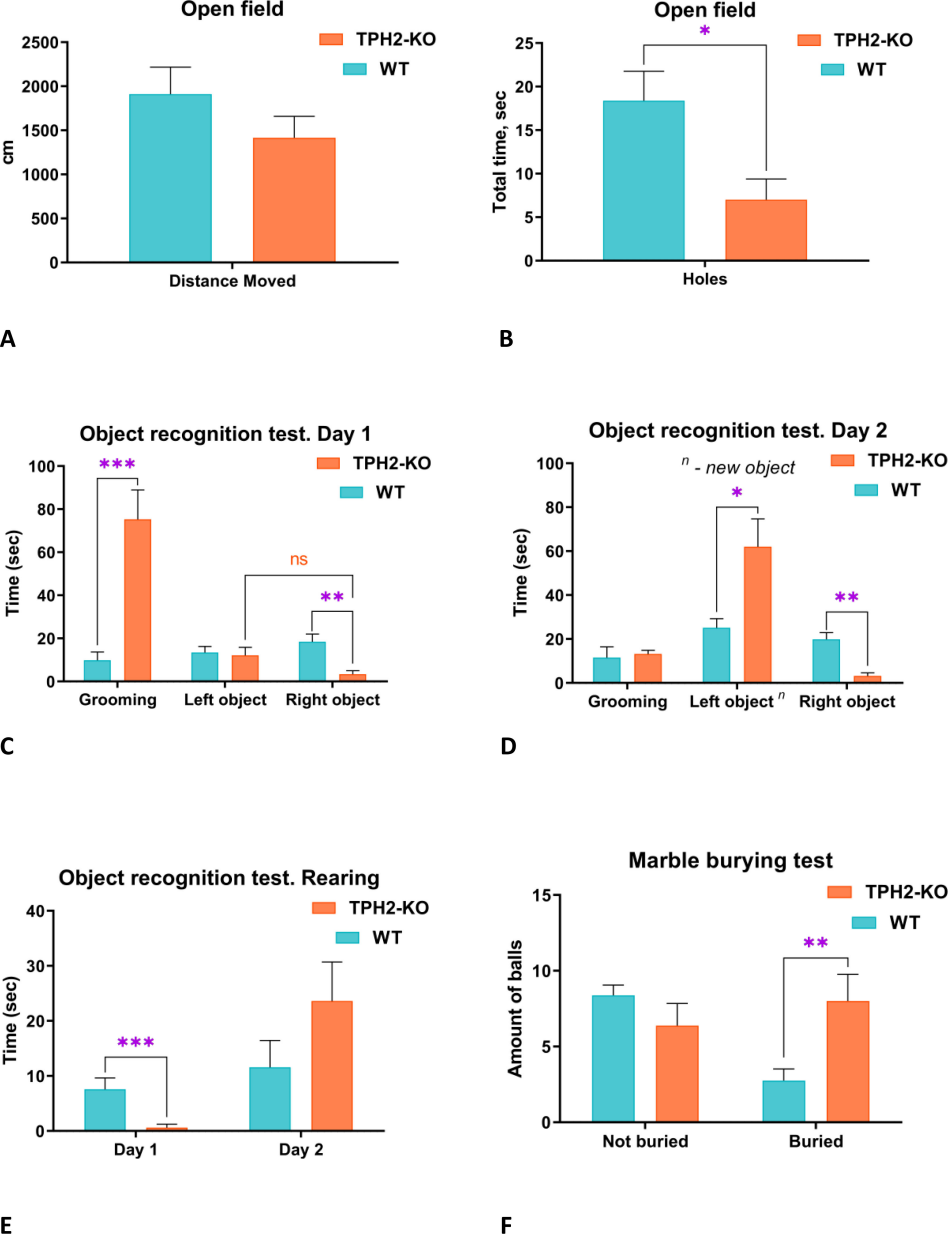
Evaluation of baseline behavioral phenotype of TPH2-KO rats. **(A, B)** TPH2-KO rats showed decreased hole exploration in circular open-field test (*p =* 0.0260). Other parameters do not reach significant differences. **(C)** In the novel object recognition test (ORT) TPH2-KO rats showed significant changes compared to WT. In front of similar objects (Day 1), TPH2-KO rats demonstrated significantly increased grooming (*p =* 0.0006) and decreased exploration of the right object (*p =* 0.0017). **(D)** Day 2 of ORT revealed increased learning of the new object (p = 0.0293), but decreased time with right one in TPH2 compared to WT rats (*p =* 0.0093). **(E)** TPH2-KO rats spent less time in rearing exploration in comparison to WT (*p =* 0.0008). **(F)** The marble burying test revealed a slightly obsessive-compulsive behavioral phenotype in the TPH2-KO group represented in a buried endpoint (*p =* 0.0065). Data are presented as mean ± SEM (n = 8). *p<0.05, **p<0.01, ***p<0.001 vs. control, Mann-Whitney U-test. WT, wild type rats.

Before the resident-intruder test, exploratory, anxiety-like and compulsive-like behaviors were evaluated in TPH2-KO rats (**Figure 2**). In the OFT, mutant rats showed decreased exploration of holes (*U =* 11, *n =* 6-8 per group, *p =* 0.0260) but otherwise unaltered anxiety-related behaviors (freezing, grooming, sniffing) or locomotor endpoints (see **Figures 2A, B** and **Supplementary Materials** for details). In the object recognition test (ORT, **Figures 2C, D**, TPH2-KO rats showed a marked preference for grooming over exploring the two identical objects (*U =* 1, *n =* 6-8 per group, *p =* 0.0006), and mostly avoided the rightward object compared to WT (*U =* 4, *n =* 6-8 per group, *p =* 0.0017) (**Figure 2C**). On Day 2, the TPH2-KO rats explored the new object significantly more than WT (*U =* 7, *n =* 6-8 per group, *p =* 0.0293). The avoidance of the rightward object persisted on Day 2, unlike WT counterparts (*U =* 4.5, *n =* 6-8 per group, *p =* 0.0093) which showed no such laterality on either day. The mutants also reared less on the identical objects in the ORT (*U =* 2.5, *n =* 6-8 per group, *p =* 0.0008) (**Figure 2E**). In the marble burying test, TPH2-KO rats exhibited increased compulsive-like tendencies (*U =* 7, *n =* 6-8 per group, *p =* 0.0065) (**Figure 2F**), which corroborates prior results in mice (47, 48).

**Figure 3:**
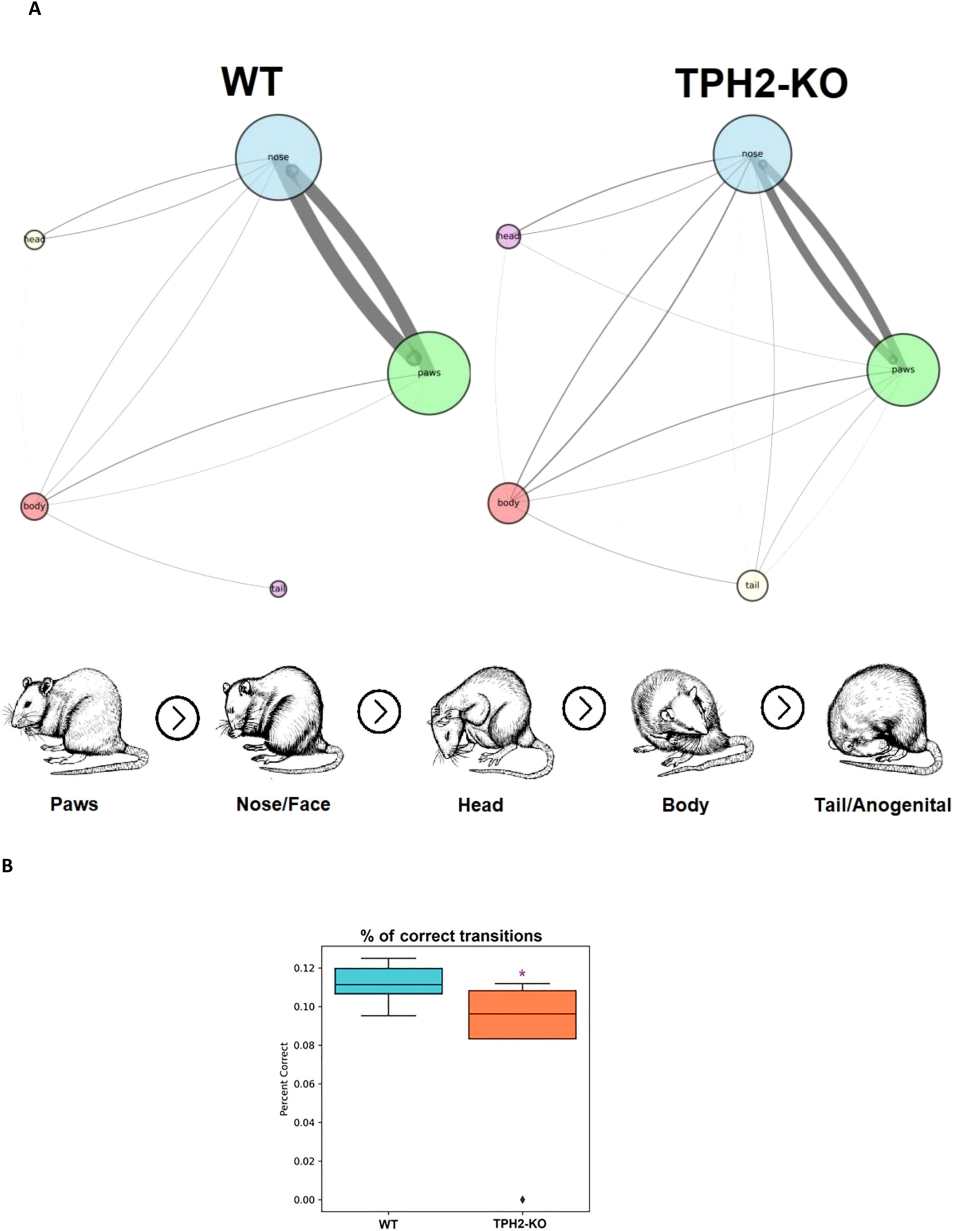
TPH2-KO rats have slightly decreased percent of correct transitions in grooming microstructure. **(A)** Comparative self-grooming microstructure analyses of TPH2 knockout (KO) rats vs. wild type (WT) control group in the grooming test (n = 8 per group). The diameter of circles and line thickness reflect mean frequency of grooming bouts or transitions, respectively. All grooming bouts, but only ‘correct’ grooming transitions adhering to the cephalo-caudal progression (paws > face > head > body > tail/genitals) were statistically assessed. **(B)** TPH2-KO rat had decreased percent of correct grooming transitions (*p =* 0.048) supporting its higher sensitivity to changes in grooming behavior. *p < 0.05 vs. control, Mann–Whitney U-test. WT, wild type control rat.

The study included an in-depth analysis of grooming microstructure, since DA signaling in the basal ganglia drives sequentially patterned behaviors such as grooming (49–52). Disrupted 5-HT synthesis, as observed in TPH2 knockout mice, also seem to be linked to altered grooming habits (44). Given the importance of DA and 5-HT in psychiatry, grooming metrics are often used as indicators of underlying psychiatric-like conditions (44). **Figures 3A, B** summarizes grooming data in TPH2-KO and WT groups, showing subtle alterations in the cephalo-caudal progression (paws > face > head > body > genitals/tail) and slightly reduced percent of correct grooming transitions (*U =* 45.5, *n =* 6-8 per group, *p =* 0.048) in the TPH2-KO group (**Figure 3B**; **Table 1**).

**Table 1:** Summary of statistical results of TPH2 knockout (TPH2-KO)-induced behavioral changes in mouse grooming test and grooming microstructure analysis.

Endpoint	WT	TPH2-KO	*U*	*p*
**Correct transitions, %**	**0.11 ± 0.01**	**0.09 ± 0.04**	**45.5**	**0.048**
Total grooming, s	27.20 ± 34.27	58.03 ± 80.72	35.0	0.659
Rostral grooming, s	20.49 ± 20.27	18.18 ± 21.21	45.0	0.723
Caudal grooming, s	6.71 ± 15.44	38.14 ± 66.04	21.0	0.090
Latency to grooming, s	331.24 ± 210.16	185.57 ± 111.75	62.0	0.178
Paws grooming bouts, n	14.78 ± 15.90	11.22 ± 14.19	45.0	0.723
Nose grooming bouts, n	15.78 ± 16.97	13.11 ± 15.90	46.5	0.626
Head grooming bouts, n	0.78 ± 1.64	1.22 ± 2.94	40.0	1.000
Body grooming bouts, n	1.56 ± 2.88	3.56 ± 5.17	28.0	0.271
Tail grooming bouts, n	0.56 ± 1.33	2.00 ± 4.56	31.5	0.370
Paws to nose transitions, n	12 ± 13.47	8 ± 10.39	46.5	0.625
Nose to head transitions, n	0.78 ± 1.64	1.11 ± 2.61	40.0	1.000
Head to body transitions, n	0.11 ± 0.33	0.22 ± 0.44	36.0	0.585
Body to tail transitions, n	0.44 ± 1.01	0.55 ± 1.01	36.5	0.694

Data for n = 8 per genotype are presented as mean ± SD with *U*- and *p*-values from Mann-Whitney U-tests.

Bold values - statistically significant changes.

### 3.2 TPH2-KO rats pursue dominance in the resident-intruder test

**Figure 4:**
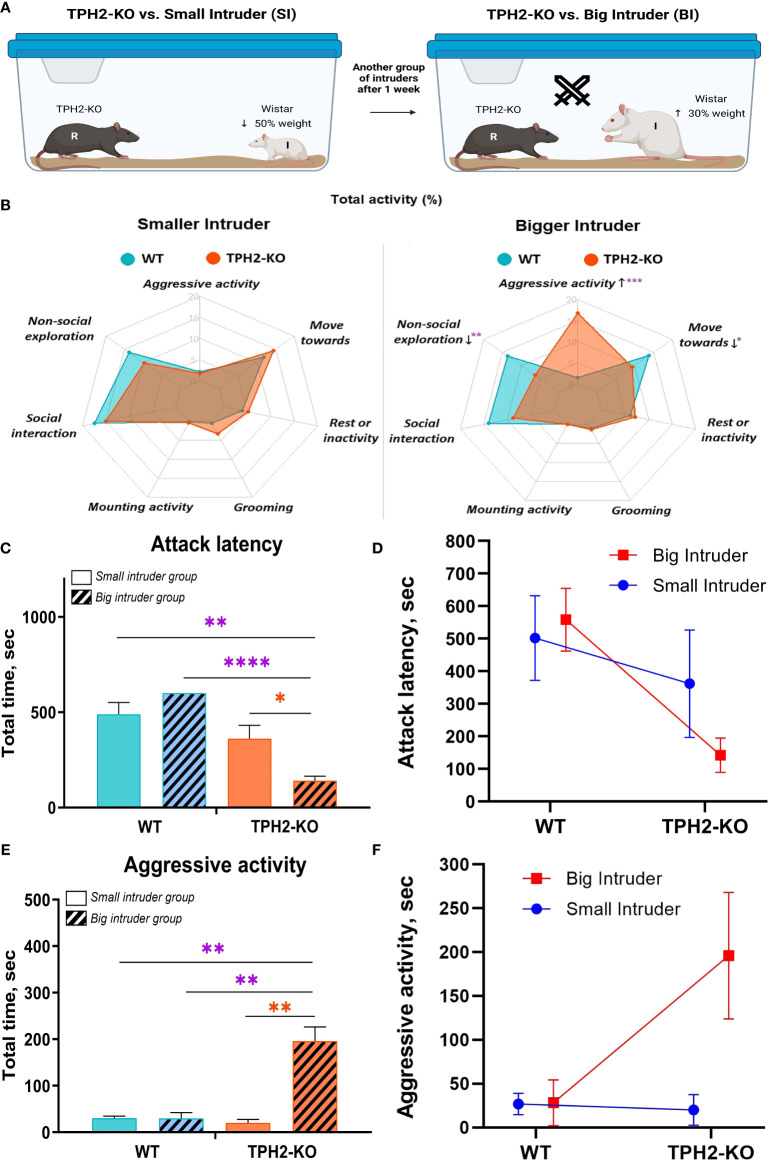
TPH2-KO rats cannot accept social defeat in the resident-intruder test. **(A)** Represents scheme of resident-intruder test with different types of intruders. TPH2-KO and WT rats opposed in front of SI and BI groups. **(B)** Radar-plot with total activity demonstrates a significant difference in TPH2-KO rats’ behavior versus the BI group. Minimal alterations were revealed in the SI group experiment. TPH2 gene knockout causes significantly increased aggressive behavior (*p =* 0.0002) in front of the larger opponent, decreased non-social exploration (*p =* 0.0031), and locomotor activity (*p =* 0.0216). **(C–F)** In the BI experiment, TPH2-KO vs. WT also demonstrated significantly decreased attack latency (*p <* 0.0001) and increased aggressive activity (*p =* 0.0029). Data are presented as mean ± SEM (n=8). *p<0.05, **p<0.01, ***p<0.01, ****p ≤ 0.0001 vs. control; Two-way repeated measures ANOVA (Factor 1 – genotype: WT/TPH2-KO; Factor 2 – intruder type: big/small) followed by Dunnett’s *post-hoc* test. WT, wild type control rats. .

To determine whether TPH2-KO aggression is motivated by the pursuit of dominance, an extended resident-intruder test was applied with two types of intruder groups (**Figure 4A**), operationalizing relative dominance with weight classes. Aggression towards smaller intruders did not significantly vary by genotype (**Figure 4B**). These conflicts were brief, as the smaller intruders were quick to assume a submissive pose, and the TPH2-KO rats typically refrained from any further aggression. With larger intruders, aggressive activity was markedly prolonged in the TPH2–KO group compared to WT (*F^Genotype^* (1, 7) *=* 25; *F^Intruder^* (1, 7) *=* 25, *p =* 0.0002), with reduced attack latency (*F^Genotype^* (1, 7) *=* 33; *F^Intruder^* (1, 7) *=* 2, *p <* 0.0001), increased ‘move-towards’ activity (*F^Genotype^* (1, 7) *=* 3; *F^Intruder^* (1, 7) *=* 3, *p =* 0.0216), and non-social exploration (*F^Genotype^* (1, 7) *=* 11; *F^Intruder^* (1, 7) *=* 2, *p =* 0.0031) (**Figures 4C–F**). Despite the bigger size of intruders, TPH2-KO rats exhibited more lateral threats (*F^Genotype^* (1, 7) *=* 31; *F^Intruder^* (1, 7) *=* 31, *p =* 00.0023) and clinch attacks (*F^Genotype^* (1, 7) *=* 19; *F^Intruder^* (1, 7) *=* 4, *p =* 0.0355). See **Supplementary Figure S2A** in **Supplementary Materials** for plots of these results.

### 3.3 TAAR1 partial agonist RO5263397 decreases social aggression in TPH2-KO rats

**Figure 5:**
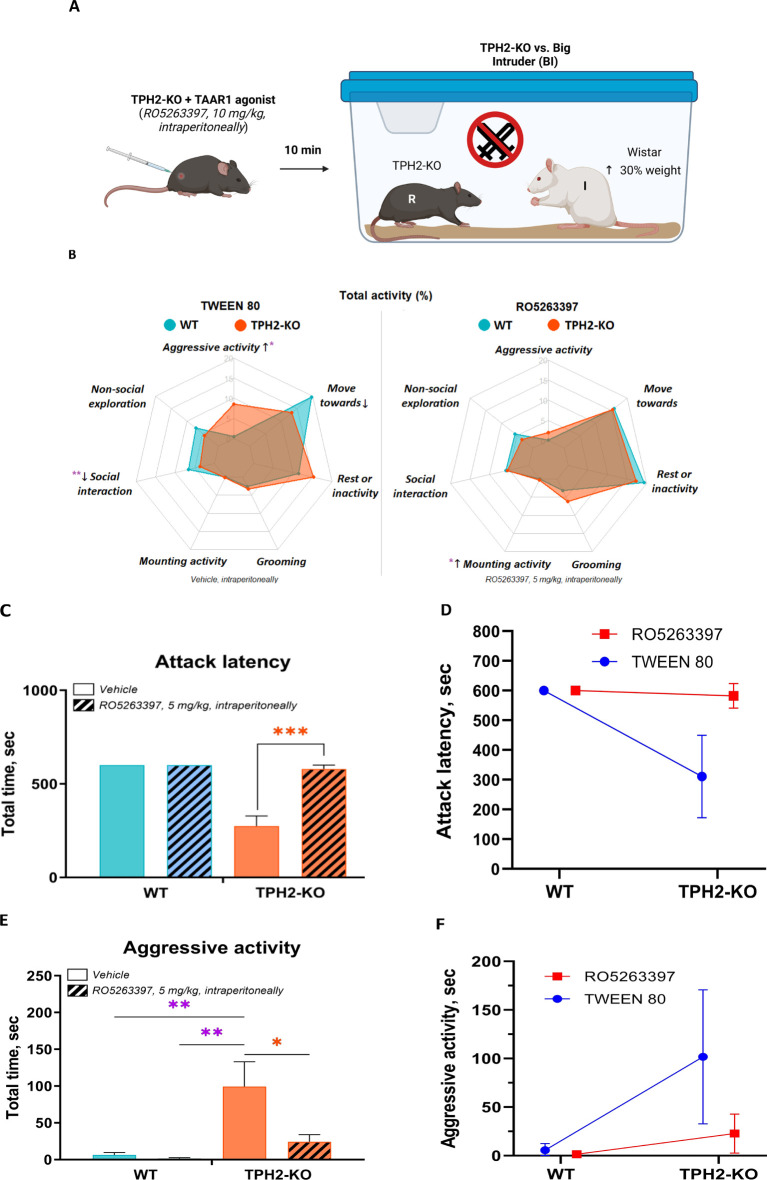
TAAR1 agonist decreases social aggression in TPH2 knockout rats. **(A)** RO5263397 (5 mg/kg) or vehicle were administered intraperitoneally 10 min before the start of the resident intruder interaction with TPH2-KO and WT rats opposed in front of the BI group. **(B)** RO5263397 normalizes the behavioral profile in the resident-intruder test, by decreasing aggressive activity (*p =* 0.0087) and slightly increasing mounting activity (*p =* 0.0338). **(C-F)** In the experiment with TPH2-KO vs. WT RO5263397 normalize alterations in attack latency and aggressive activity endpoints. Data are presented as mean ± SEM (n = 8). *p<0.05, **p<0.01, ***p<0.01 vs. control; Two-way repeated measures ANOVA (Factor 1 – genotype: WT/TPH2-KO; Factor 2 - drug effect: RO5263397/TWEEN80) followed by Dunnett’s *post-hoc* test. WT, wild type control rats.

Next, the TAAR1 agonist RO5263397 was administered to test whether TAAR1 activity attenuates the aggression revealed in the prior experiment (**Figure 5A**). Ten minutes after intraperitoneal injection, the TAAR1 agonist fully rescued the behavior of TPH2-KO rats (**Figure 5B**). Vehicle (10% buffered Tween 80) treated mutants showed decreased attack latency compared to those treated with RO5263397 [*F^Genotype^* (1, 7) *=* 34; *F^Drug^* (1, 7) *=* 29, *p =* 0.0005], which became indistinguishable from WT. Aggressive activity also decreased markedly [*F^Genotype^* (1, 7) *=* 26; *F^Drug^* (1, 7) *=* 11, *p =* 0.0087], with a slight increase in mounting activity [*F^Genotype^* (1, 7) *=* 9; *F^Drug^* (1, 7) *=* 2, *p =* 0.0338] (**Figures 5B–F**).

## 4 Discussion

The present study corroborates in rats that 5-HT depletion alters exploratory behaviors and increases aggression (21–23). The resident-intruder test results refine the current understanding of 5-HT’s involvement in rodent aggression by explicitly linking it to the drive to advance in social dominance hierarchies. Indeed, when confronted with a smaller intruder, the 5-HT-deficient rats behave as WT littermates do, whereas when the intruder is larger, the mutant rats prolong their aggressive efforts, refusing to submit. Since rats form social hierarchies based on body weight (35), dominating smaller rats does not advance social rank, but dominating larger ones does. Thus, these results show that 5-HT depletion selectively increases aggression to capitalize on opportunities to advance the rat’s social status. Most importantly, the activation of TAAR1 abolishes this abnormal aggression in mutant rats, a finding of both clinical and social significance (3). The other behavioral assays recapitulated features of compulsive behavior previously established TPH2-KO mice (47), confirming the conservation of mechanisms.

The effect of 5-HT depletion on aggression is well-replicated in TPH2-KO mice (19, 20), and the results herein corroborate this phenotype in rats, indicating that it is conserved across species. These statements pertain entirely to males, as female rats tend not to challenge conspecifics of equal or larger size (46). The 5-HT depletion idea also applies to primates, as reduced 5-HT and its metabolites are evident in highly aggressive nonhuman primates (25, 53), and lower 5-HT signaling is correlated with aggressive tendencies in humans (26). Indeed, central levels of 5-HT and its main metabolite are lower in individuals diagnosed with antisocial personality disorder (25), a diagnosis strongly associated with aggression (54). Brain samples from suicide victims also show increased expression of dorsal raphe 5-HT_1A_ receptors, implying reduced serotonergic signaling given its inhibitory autoreceptor function (24). Taken together, these findings imply that 5-HT depletion can trigger aggression both inwardly (i.e., self-harm or suicide), and outwardly (i.e., emotional abuse or physical attacks); this aligns well with experimental results on 5-HT depletion in humans (55).

The SSRIs are efficacious in many conditions other than depression (56). Given their mechanism of action, this transdiagnostic efficacy implies that many patients experience their symptoms in part due to 5-HT depletion. For instance, the undue feelings of guilt and shame that are common in depression can be induced by 5-HT depletion in humans (55). Genetic predispositions to such disorders also implicate the serotonergic system. The depression-related SNP (R439H) in the human *Tph2* gene greatly diminishes 5-HT production, and chronic SSRI treatment exacerbates this depletion in humanized mouse carriers of R439H (13). This effect may not be unique, as other mutations may perturb the same functions, and thus lead to the same depletion under chronic SSRI use (16, 57, 58). It remains unknown whether this paradoxical effect contributes to SSRI-related violence. However, as mentioned in the introduction, human studies have established that perceptions of one’s social rank motivate violence in young people. The present study concords with this, suggesting that the aggression of the TPH2-KO rat is motivated by a drive to advance in social rank.

The most important finding here is that this abnormal form of aggression is abolished by TAAR1 activation. This is particularly promising because TAAR1 is known to support cognitive control (59–61). Deficits in cognitive control are not only associated with aggression and self-harm, they are also transdiagnostic (62), which suggests the future potential of TAAR1 agonists as common adjuncts in psychiatry. A burgeoning literature also establishes the biological plausibility of TAAR1 agonists addressing both mania and depression (60, 63), in addition to anxiety (64), psychosis (65), and features of ADHD (60, 66). With respect to mechanism, it bears mentioning that different TAAR1 agonists can either inhibit or stimulate activity of serotonergic neurons of the dorsal raphe nucleus (DRN) and the dopaminergic neurons of the ventral tegmental area (VTA) (39, 67). Both of these areas are known to have reciprocal projections with the anterior cingulate cortex (ACC) and the mPFC (68), a crucial hub in the top-down regulation of aggression (27).

Since TAAR1 is expressed both in the mPFC and several regions that project to it (60), there are several possible pathways by which TAAR1 activation can limit aggression. The effects of TAAR1 on aggression can primarily occur through direct glutamatergic effects in the mPFC, as TAAR1 modulates both receptors and transporters for glutamate (39, 69, 70). It has also been shown that sulpiride, a selective D2 antagonist, diminishes the aggressive social dominance of high ranking macaques (53). TAAR1 is known to desensitize D2 (71); a kind of soft ‘D2 blockade’ which may be the basis of TAAR1 agonism’s antipsychotic effects (72). In the case of the D2 autoreceptor, TAAR1-mediated desensitization should thus lead to greater propagation of DA signals across networks, and the opposite effect would apply in the case of postsynaptic D2. It is unclear which of these mechanisms dominated the results herein, but DA levels are associated with social dominance dynamics (73). DA promotes aggression through the VTA’s projection to the lateral septum (74); TAAR1 may have inhibited this pathway.

Unlike those of D2, the possible contributions of 5-HT_1A_ are harder to specify. The absence of 5-HT in TPH2-KO rats implies that 5-HT_1A_ should be inactive. Many experiments have nevertheless shown that 5-HT_1A_ is constitutively active (75), even in *ex vivo* preparations from rats (76). Intriguingly, the 5-HT_1A_ agonist befiradol diminishes the increased aggression of TPH2-KO rats (21). The authors of this study specifically attributed the increased aggression in TPH2-KO rats to decreased sensitivity at 5-HT_1A_. As it happens, TAAR1 activation sensitizes 5-HT_1A_ (39), but it is unknown whether the sensitized conformation favors ligand binding or constitutive activity. Based on these reports, the contribution of TAAR1-mediated 5-HT_1A_ sensitization to the attenuation of aggression in TPH2-KO rats cannot be excluded. In summary, TAAR1 may affect the mPFC through glutamate, dopamine, and serotonin to abolish abnormal aggression. These mechanisms establish the biological plausibility of TAAR1 agonists as a treatment for pathological aggression, and the outcomes of the resident-intruder tests are a proof of this concept.

## 5 Future directions

Despite brilliant work on the neurodevelopment of the 5-HT system (see 77 for a representative review), there has not yet been a clear definition of critical or vulnerability periods for the role of 5-HT in social aggression or self-harm. There is also the underexplored possibility of serotonin syndrome leading to aggression by paradoxical effects (e.g., hyperactivation of 5-HT_1A_ autoreceptors). Equally interesting is the question of dietary tryptophan deprivation: To what extent does low tryptophan intake contribute to uncharacteristic acts of aggression or self-harm? This is particularly interesting *vis a vis* co-morbidity in eating disorders; many such cases are female. The age- and sex-differences evident in aggression and self-harm may also be contingent upon personality; this is worth confirming for more well-informed clinical practices in the future. Furthermore, TAAR1 is the only member of its family that has been explicitly studied with respect to aggression; other TAARs remain obscure. Difficult as they are to investigate, these possibilities are worthwhile given their relevance to the devastating burdens of aggression and self-harm.

## 6 Conclusion

Aggression and self-harm are major sociomedical problems that disproportionately occur in young people. Independent studies attribute this to a fixation on social status cues in adolescence and young adulthood. Multiple lines of evidence link 5-HT depletion to antisocial behavior across species, and the present study shows in middle-aged rats that this effect is motivated by gains in social status. Crucially, the TAAR1 agonist RO5263397 selectively abolishes this abnormal aggression in 5-HT-depleted rats. It also bears mentioning that recent independent results confirm this finding without TPH2-KO, showing that the same TAAR1 agonist as well as a 5-HT_1B_ agonist also attenuate another form of abnormal aggression in the resident-intruder paradigm (36). These results are in line with several known interactions between TAAR1, glutamate, and canonical monoamine receptors. This establishes the biological plausibility of TAAR1 agonism as a treatment for aggressive behavior. Further study of TAAR-modulating compounds is thus warranted, especially since these mechanisms are largely conserved in primates. In this light, the efficacy of TAAR1 agonists in aggression may translate to the clinic.

## Data Availability

The original contributions presented in the study are included in the article/**Supplementary Material**. Further inquiries can be directed to the corresponding author.
