# Supplementary material for: Trace amine-associated receptor 1 agonist reduces aggression in brain serotonin-deficient tryptophan hydroxylase 2 knockout rats
Source: Front Psychiatry. 2024 Dec 19;15:1484925. doi: 10.3389/fpsyt.2024.1484925 (PMC11693706; doi:10.3389/fpsyt.2024.1484925)

Supplementary Figure 1. Full results for open field test.

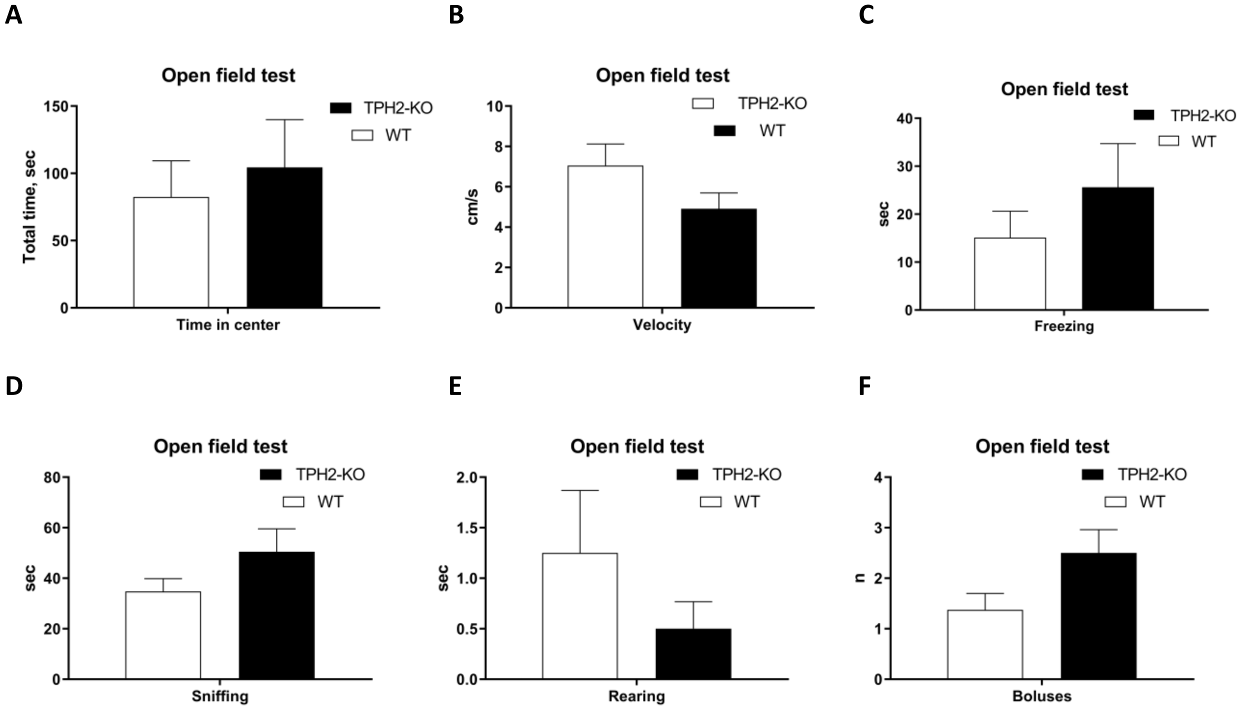

Supplementary Figure 2. Full visualization of extended resident-intruder test with different types of intruders.

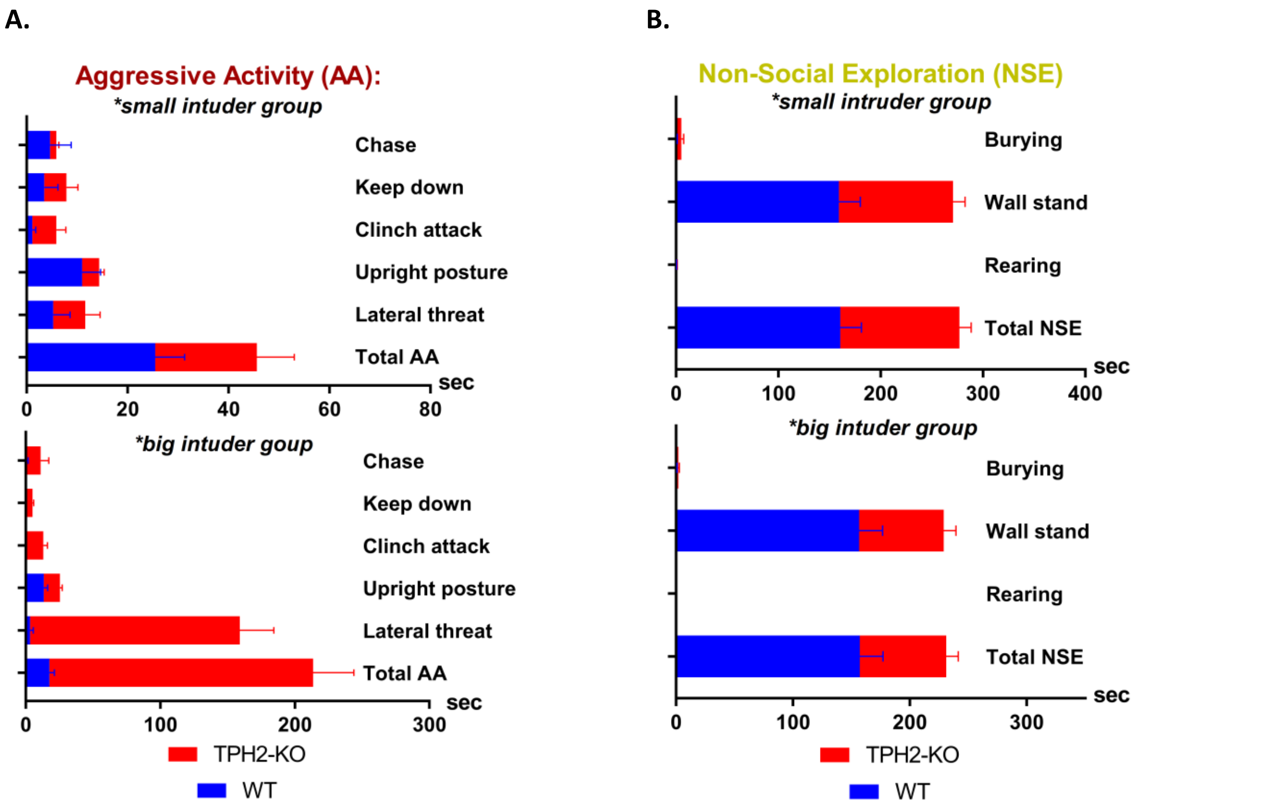

C.

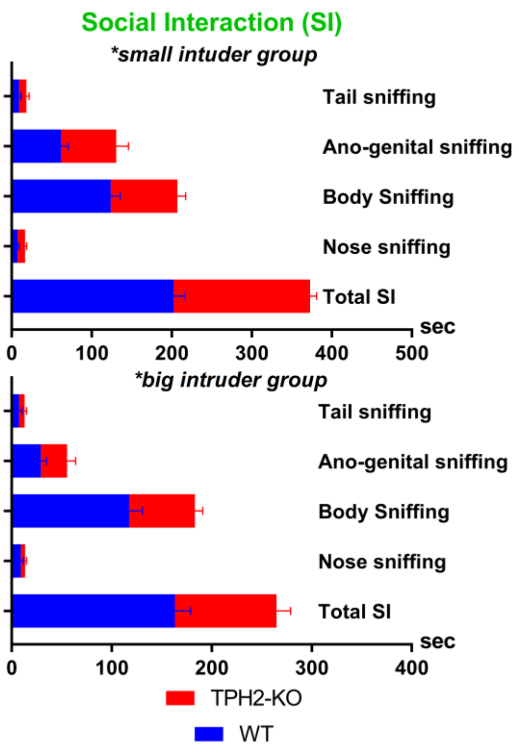

D.

Endpoint / P value*	Small: WT vs. Big: TPH2-KO	Small: TPH2-KO vs. Big: WT	Small: TPH2-KO vs. Big: TPH2-KO	Big: WT vs. Big: TPH2-KO
**Aggressive activity**	*****P = 0.0002**	**NS**	*****P = 0.0002**	*****P = 0.0002**
Lateral threat, s	**P = 0.0036	NS	**P = 0.0023	**P = 0.0023
Upright posture, s	NS	*P = 0.0285	*P = 0.0122	NS
Clinch attack, s	**P = 0.0056	NS	*P = 0.0355	**P = 0.0034
Keep down, s	NS	NS	NS	***P = 0.0008
Chase, s	NS	NS	NS	NS
**Non-Social Exploration**	****P = 0.0027**	**NS**	**NS**	****P = 0.0031**
Rearing, s	NS	NS	NS	NS
Wall stand, s	*P = 0.0495	NS	NS	NS
Burying, s	NS	NS	NS	NS
**Social Interaction**	****P = 0.0039**	**NS**	***P = 0.0379**	**NS**
Nose sniffing, s	NS	NS	NS	NS
Body sniffing, s	**P = 0.0013	NS	NS	NS
Ano-genital sniffing, s	NS	NS	NS	NS
Tail sniffing, s	NS	NS	NS	NS

Small: *WT* vs. Small: *TPH2-KO *and* **Small: *WT* vs. Big: *WT*** *– all endpoints* ***NS***

E.

WT (Resident)	Weight (g)	TPH2-KO (Resident)	Weight (g)	Small (Intruder)	Weight (g)	Big (Intruder)	Weight (g)
**2688 (I-A)**	297	**2695 (IV-A)**	305	**I**	154	**A**	465
**2711 (IV-A)**	312	**2694 (III-C)**	331	**II**	163	**B**	425
**2699 (I-A)**	302	**2690 (II-B)**	338	**III**	150	**C**	433
**2681 (II-B)**	347	**2704 (II-B)**	285	**IV**	150	**D**	380
**2689 (II-B)**	305	**2705 (I-C)**	308				
**2698 (II-B)**	337	**2706 (I-A)**	330				
**2710 (IV-A)**	279	**2707 (III-C)**	320				
**2712 (IV-C)**	316	**2685 (I-A)**	321				

F

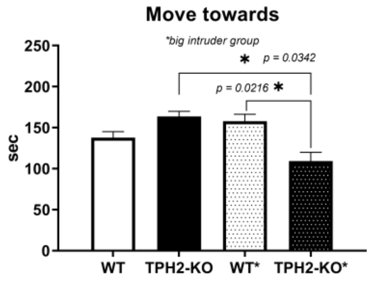

G

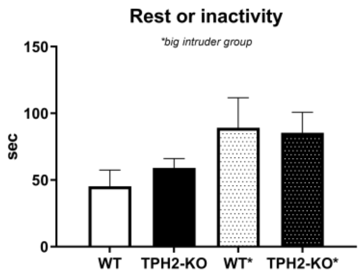

H

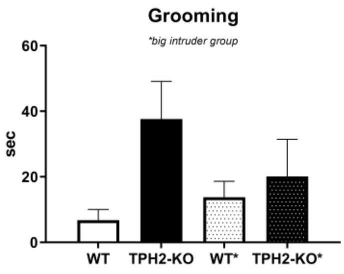

I

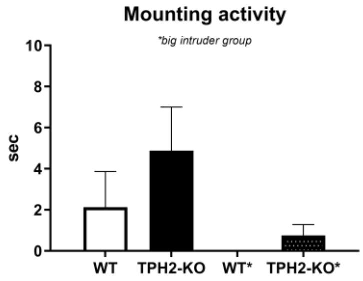

J

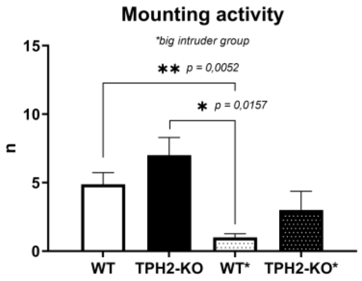

K

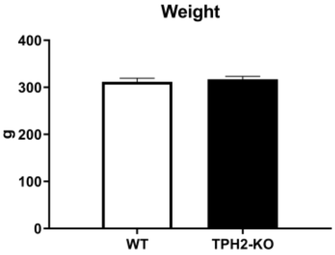

Supplementary Figure 3. Full visualization of resident-intruder test with RO5263397 administration.

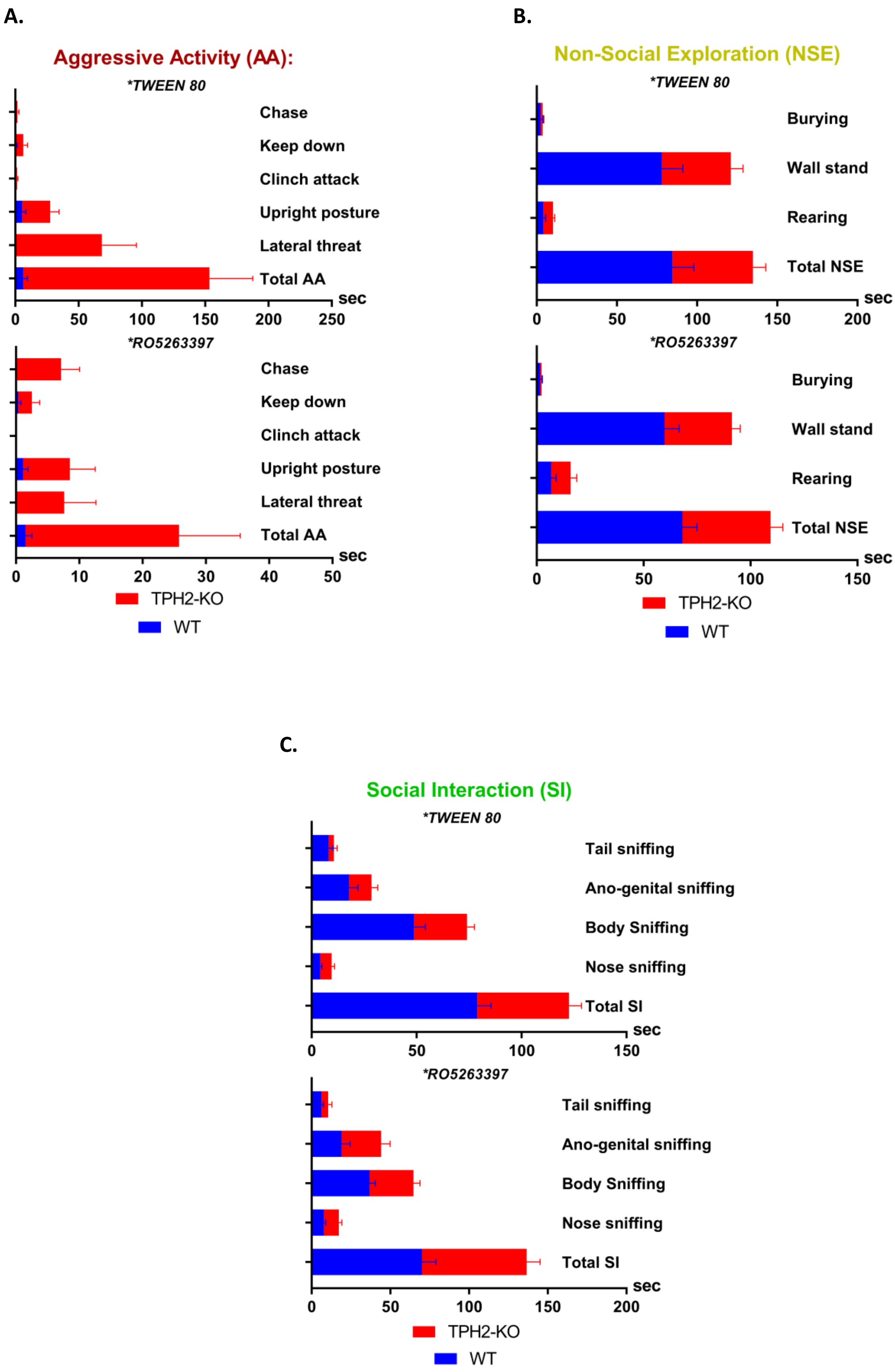

D.

Endpoint / P value*	TWEEN: WT vs. TWEEN: TPH2-KO	TWEEN: WT vs. RO: TPH2-KO	TWEEN: TPH2-KO vs. RO: WT	TWEEN: TPH2-KO vs. RO: TPH2-KO
**Aggressive activity**	****P = 0.0087**	**NS**	****P = 0.0073**	****P = 0.0177**
Lateral threat, s	*P = 0.0228	NS	*P = 0.0228	*P = 0.0361
Upright posture, s	NS	NS	*P = 0.0203	NS
Clinch attack, s	**P = 0.0048	NS	**P = 0.0048	**P = 0.0048
Keep down, s	NS	NS	NS	NS
Chase, s	NS	NS	NS	NS
**Non-Social Exploration**	**NS**	**NS**	**NS**	**NS**
Rearing, s	NS	NS	NS	NS
Wall stand, s	NS	NS	NS	NS
Burying, s	NS	NS	NS	NS
**Social Interaction**	****P = 0.0058**	**NS**	***P = 0.0260**	***P = 0.0484**
Nose sniffing, s	NS	NS	NS	NS
Body sniffing, s	*P = 0.0390	**P = 0.0064	NS	NS
Ano-genital sniffing, s	NS	NS	NS	NS
Tail sniffing, s	NS	NS	NS	NS

****Tween: WT vs. RO: WT* and *RO: WT vs. RO: TPH2-KO* – *all endpoints* ***NS***

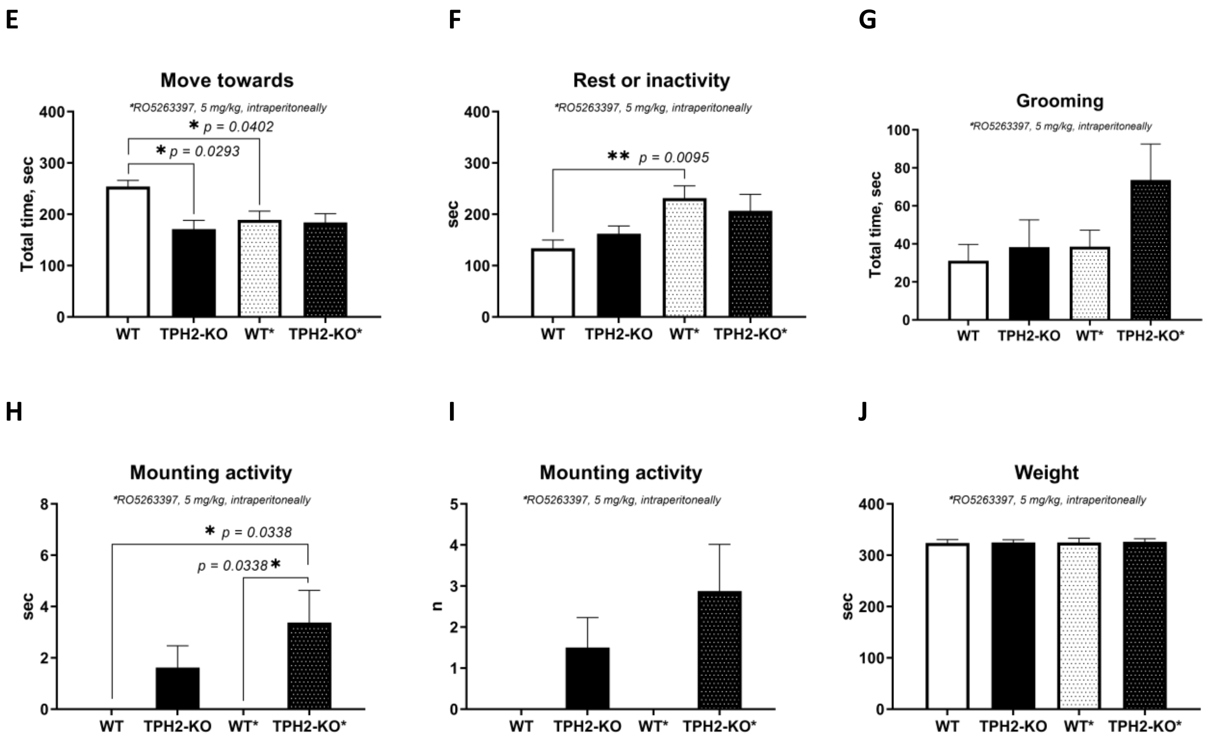

Supplement: Supplementary file 1 [file DataSheet1.pdf]
